# Hospital Readmission Reduction Program Penalties for Hospitals With High Medicare Advantage Penetration

**DOI:** 10.1001/jamanetworkopen.2025.54972

**Published:** 2026-01-22

**Authors:** Zoey Chopra, Andrew M. Ryan, Geoffrey J. Hoffman

**Affiliations:** 1 Department of Economics, University of Michigan, Ann Arbor; 2 Population Studies Center, Institute for Social Research, University of Michigan, Ann Arbor; 3 University of Michigan Medical School, Ann Arbor; 4 Center for Advancing Health Policy Through Research, Brown University, Providence, Rhode Island; 5 Department of Health Services, Policy, and Practice, Brown University, Providence, Rhode Island; 6 Institute for Healthcare Policy and Innovation, University of Michigan, Ann Arbor; 7 Department of Systems, Populations and Leadership, University of Michigan School of Nursing, Ann Arbor

## Abstract

**Importance:**

Since 2012, the Hospital Readmissions Reduction Program (HRRP) has penalized hospitals for excess, risk-adjusted 30 day readmissions among traditional Medicare (TM) beneficiaries. While risk adjustment may address observable differences in patient severity, it cannot account for unobservable differences. Medicare Advantage (MA) enrollment has continued to increase, and MA beneficiaries have been found to be both observably and unobservably healthier than their TM counterparts. Because relatively lower-severity patients are increasingly likely to enroll in MA, hospitals with higher MA penetration may have unobservably higher-severity TM patients, resulting in higher-than-estimated readmission risk and excessive HRRP penalties.

**Objective:**

To determine whether unobserved selection, as proxied by MA penetration, could be associated with distorted HRRP penalties and how associations may be moderated by peer grouping, which was incorporated into HRRP’s 2019 revision to penalty calculations.

**Design, Setting, and Participants:**

This retrospective cohort study included hospitals serving Medicare beneficiaries from fiscal years 2019 to 2022 for 6 HRRP-targeted conditions, including acute myocardial infarction, chronic obstructive pulmonary disease, heart failure, pneumonia, coronary artery bypass graft surgery, and elective primary total hip or knee arthroplasty. Data were analyzed from January 2024 to October 2025.

**Exposure:**

MA penetration at the hospital-year level.

**Main Outcomes and Measures:**

Excess readmission ratios (ERRs) and calculated HRRP penalties at the hospital-year level. To test whether HRRP penalties could have been distorted by unobserved selection, the association between the share of hospital admissions for MA patients (MA penetration) and excess readmission ratios (ERRs) for all patients was estimated, controlling for county-level variation and hospital-level covariates. The ERRs were rescaled by MA penetration to account for unobserved selection, and the rescaled ERRs were used to reestimate HRRP penalties under non–peer grouping and peer grouping paradigms.

**Results:**

This study included 3203 hospitals and 12 135 hospital-years. After adjusting for MA penetration, estimates indicated that hospitals in the first quintile of MA penetration would be penalized by a mean (SD) of $30 736 ($24 819.75) more, while hospitals in the fifth quintile would be penalized by a mean (SD) of approximately $26 915 ($42 017.23) less. Peer grouping does not mitigate these penalty distortions. Across hospitals, penalty redistributions would amount to $284 to $297 million annually.

**Conclusions and Relevance:**

The findings of this study suggest that including MA penetration explicitly in risk adjustment or in peer group definitions may dampen distortions from unobservable patient severity in HRRP penalty calculations.

## Key Points

**Question:**

Is unobserved selection associated with distortion in Hospital Readmissions Reduction Program (HRRP) penalties?

**Findings:**

This cohort study of 3203 hospitals found that after adjusting for unobserved selection as proxied by Medicare Advantage (MA) penetration, hospitals in the first quintile of MA penetration would be penalized $30 736 more while hospitals in the fifth quintile would be penalized $26 915 less. These distortions were observed with and without peer grouping; across hospitals, penalty redistributions would amount to $284 million to $297 million annually.

**Meaning:**

These findings suggest that hospitals with higher MA penetration have inflated HRRP penalties because risk-adjustment cannot account for unobservable differences between MA and non-MA beneficiaries; accounting for MA penetration explicitly may dampen distortions from unobservable patient severity in HRRP penalty calculations.

## Teaser

This cohort study assesses whether observed selection distorted Hospital Readmissions Reduction Program penalties and how this may be moderated by peer grouping.

## Introduction

In October 2012, the Centers for Medicare & Medicaid Services (CMS) implemented the Hospital Readmissions Reduction Program (HRRP) to penalize hospitals for excess 30-day readmissions among traditional Medicare (TM) beneficiaries.^1^ HRRP relies on risk adjustment involving observable patient diagnoses to profile hospital-specific readmission performance.^2^ However, if patients with unobservably greater disease severity cluster within hospitals, risk adjustment based only on observable characteristics may compromise the accuracy of HRRP quality profiles and distort penalties.

Concerns about unobserved severity are heightened by considerable growth in Medicare Advantage (MA) enrollment, which increased from 19% in 2007 to 54% in 2025^3^ with substantial regional variation.^4,5^ If MA beneficiaries were only healthier than TM beneficiaries across observable characteristics, geographic differences in MA penetration should not affect HRRP hospital quality profiles, since current risk adjustment methods account for measurable patient severity. However, recent evidence has found MA beneficiaries to be both observably and unobservably healthier than TM counterparts, reflecting favorable selection of enrollees into MA.^6,7,8,9,10,11,12^ As MA penetration increases within an area, TM loses its healthier enrollees to MA and maintains a sicker residual TM population. Therefore, uneven changes in MA penetration yield unobservable differences in TM patient severity not captured by HRRP risk adjustment.

### HRRP Risk Adjustment and Hospital Profiling

From its inception in October 2012 through September 2018, HRRP calculated penalties by comparing individual risk-adjusted hospital performance to the national average.^13^ Beginning in fiscal year (FY) 2019, penalty calculations have incorporated peer grouping, which changes the reference standard to the median peer group hospital (ie, median readmission performance of hospitals with similar proportions of dually eligible [DE] beneficiaries). Peer grouping should help account for hospital differences in unobserved patient socioeconomic status (SES) and associated health risks and has been found to redistribute HRRP penalties away from hospitals with lower SES patient populations.^14,15^ However, because DE share only partially captures SES-associated health risk, peer grouping is unlikely to comprehensively address between-hospital differences in unobserved severity.^16^

Given increasing differences in unobserved severity between MA and TM, MA penetration—to the extent it can proxy for these unobservable differences—may have implications for the accuracy of HRRP penalties. Because MA beneficiaries are unobservably healthier than TM counterparts on average, hospitals with greater MA beneficiary shares likely have unobservably higher-severity TM populations. For these hospitals, TM readmission rates may be higher than expected after only accounting for observable severity in risk adjustment. Conversely, hospitals with lower MA penetration are relatively more likely to have an unobservably healthier TM population and lower than expected TM readmission rates. Because HRRP penalizes higher than expected TM readmission rates, differences in MA penetration may yield larger penalties for hospitals with higher MA penetration and smaller penalties for hospitals with lower MA penetration, even absent differences in readmission performance. Failing to consider differences in unobservable risk may yield sizable distortions in the magnitude and distribution of HRRP penalties. We investigated the association between unobserved severity and HRRP by quantifying HRRP penalty distortions from unobserved severity and examining how peer grouping moderates these associations.

## Methods

This cohort study followed the Strengthening the Reporting of Observational Studies in Epidemiology (STROBE) reporting guideline. This study was exempt by the University of Michigan institutional review board because it was secondary research for which consent was not required.

### Key Variables and Data Sources

Study outcomes included HRRP excess readmission ratios (ERRs), adjustment factors, and penalties at the hospital-year level. We used publicly available FY 2019 to 2022 HRRP Supplemental Data Files^17^ to extract CMS-calculated, hospital-specific ERRs involving TM beneficiaries discharged during each performance period for all 6 HRRP-targeted conditions: acute myocardial infarction (AMI), chronic obstructive pulmonary disease (COPD), heart failure, pneumonia, coronary artery bypass graft surgery (CABG), and elective primary total hip or knee arthroplasty (THKA). Because HRRP typically uses 3-year lookback periods to compute penalties for a given FY (eg, FY 2021 penalties are calculated using FY 2017 to 2019 performance data), to align with FY 2019 to 2022 penalty periods, we calculated hospital-level average MA penetration for each performance period using 100% MedPAR 2014 to 2020 files, which cover 89% of MA hospitalizations.^18^ We characterized hospital utilization using 100% MedPAR 2019 and 2020 files to estimate hospital-specific penalties. We used FY 2019 to 2022 CMS Impact Files to determine hospital-level covariates and Agency for Healthcare Research and Quality 2015 to 2019 Social Determinants of Health files to capture county-level characteristics. Methods for data alignment may be found in eTable 1 in Supplement 1, and details of data structure, in eMethods 1 in Supplement 1.

### Statistical Analysis

CMS computed condition-specific ERRs as ratios of predicted to expected readmissions and were estimated using hierarchical generalized linear models that adjust for demographics and condition categories but not hospital or area-level characteristics. Adjustment factors (ie, percentage reductions in per-discharge Medicare reimbursements, capped at 3%) and penalties ($) are a function of ERR-related spending across all 6 HRRP conditions relative to total discharge spending.^13^ Hospitals with excess readmissions (ERR of more than 1) for 1 or more conditions are penalized.

To test whether HRRP penalties may be distorted by unobserved selection, we rescaled ERRs after accounting for hospital-level MA penetration, then rescaled adjustment factors and penalties and compared them to unscaled counterparts. We proceeded in 3 steps.

First, we regressed the share of MA hospital admissions (MA penetration) on total condition-specific ERRs (eMethods 2 in Supplement 1). In these specifications, we included county fixed effects and several hospital-level covariates, including safety-net status, teaching status, and bed size. Following Herrin et al,^19^ we also adjusted for several time-varying, county-level characteristics, including density of home health agencies and population shares 65 years and older, never married, with less than a high school education, unemployed, and in poverty. These variables have been associated with measured readmission performance (posited to reflect compositional differences in patient population risk) and helped us control for hospital quality differences.^20,21,22^ Concerns about secular trends driving readmissions are mitigated by CMS’ use of 3-year lookback periods to measure readmission performance, and CMS’ conditioning on admissions, which likely follow the same trends. Concerns about pandemic effects are mitigated, because CMS stopped the FY 2022 lookback period in December 2019 rather than June 2020 (eTable 1 in Supplement 1). Models were weighted by condition-specific volume across performance periods. Second, after determining associations between MA penetration and original condition-specific ERRs, we calculated predicted ERRs as risk-adjusted ERRs for each condition-hospital-year, expected ERRs as risk-adjusted ERRs for each condition-year across hospitals, and rescaled ERRs as original ERRs + expected ERRs − predicted ERRs (eMethods 2 in Supplement 1). Third, following HRRP methodology, but replacing original ERRs with rescaled ERRs, we reestimated adjustment factors and penalties under both non–peer grouping and peer grouping paradigms (eMethods 2 in Supplement 1).

Statistical significance was set at *P* < .05, and all tests were 2-sided. Data were analyzed from January 2024 to October 2025 using Stata version 18.0 (StataCorp).

## Results

**Figure 1.**
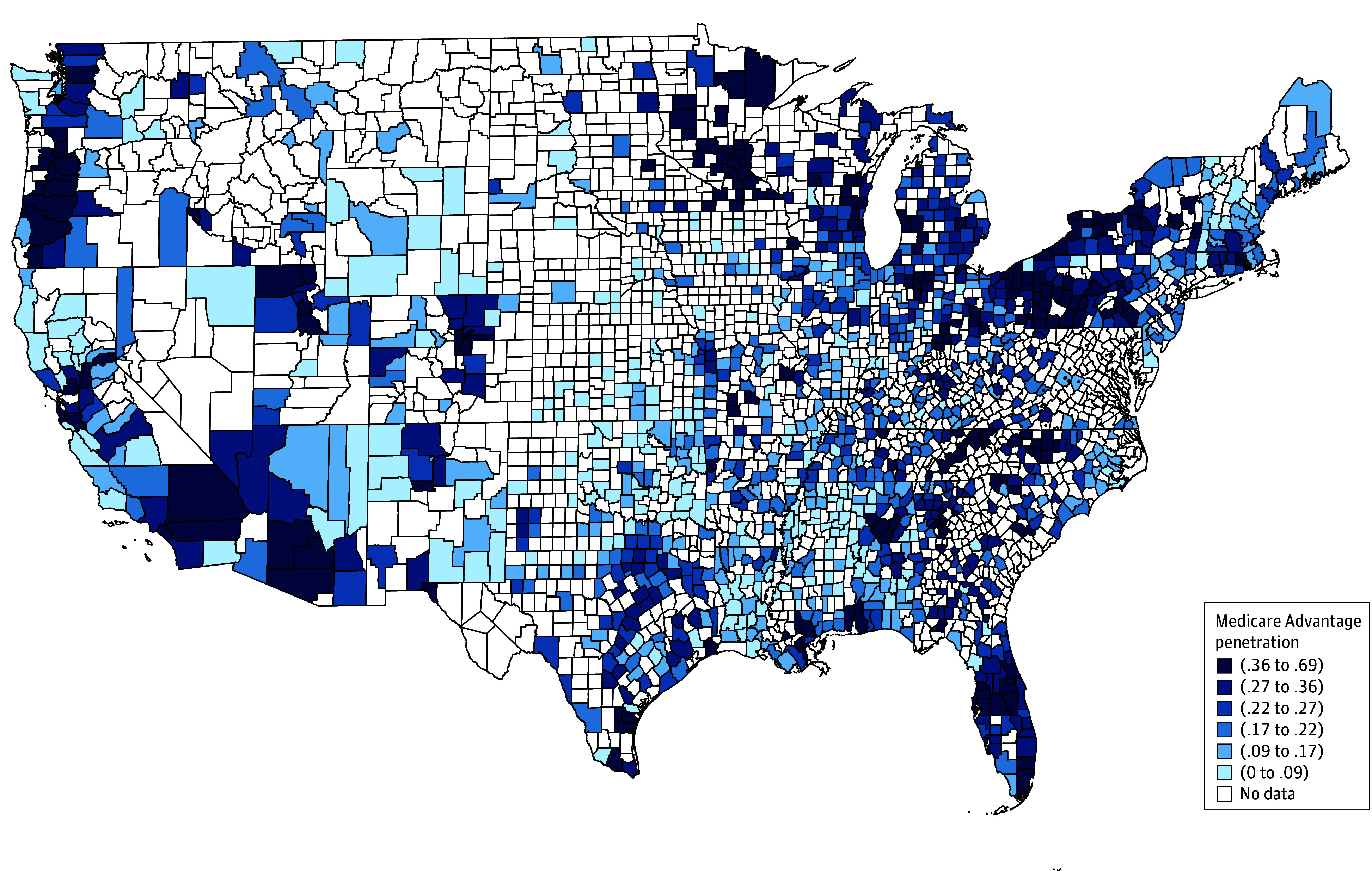
Geographic Distribution of Hospital-Level Medicare Advantage Penetration by Quintile Map discretizes mean within-hospital Medicare Advantage (MA) penetration across sample years into quintiles. Alaska (first quintile) and Hawaii (4th quintile) are omitted from the map but included in the sample. The values in the key are proportions.

Our study included 3203 hospitals and 12 135 hospital-years (Table). Hospitals with high MA penetration (fifth quintile, Q5) were more often larger, teaching, nonprofit, and urban, relative to hospitals with low MA penetration (first quintile, Q1). Mean ERRs were consistently higher (ie, more excess readmissions) in Q5 hospitals across HRRP conditions. Over our study period, mean (SD) MA penetration increased from 25.3% (16.3%) to 29.9% (15.7%) across hospitals, with noticeable hospital-level variation (Figure 1 and eTable 2 in Supplement 1).

**Table.** Hospital Characteristics in Counties With Low and High Medicare Advantage Penetration in 2019^a^

Characteristic	Hospitals, No. (%)		
	Overall (all quintiles)	Low penetration (first quintile)	High penetration (fifth quintile)
Count			
Hospitals	3165 (100)	559 (17.6)	628 (19.8)
Counties	1486 (100)	125 (8.4)	93 (6.3)
MA penetration, mean (SD), %			
Hospital-level	25.3 (16.3)	4.0 (3.5)	49.6 (12.2)
County-level	30.6 (13.9)	17.6 (12.9)	47.9 (8.2)
Total episode volume, mean (SD)			
AMI	164.5 (206.0)	92.6 (164.5)	141.8 (156.9)
COPD	256.0 (229.5)	164.3 (203.8)	203.4 (178.2)
Heart failure	360.6 (358.9)	208.0 (284.2)	301.7 (277.7)
Pneumonia	410.0 (358.2)	278.8 (328.2)	334.0 (284.4)
CABG	114.0 (103.6)	119.1 (103.6)	75.4 (63.8)
THKA	341.4 (457.6)	338.8 (669.8)	247.0 (325.4)
Bed size			
1-50	656 (20.7)	261 (47.1)	64 (10.2)
51-100	548 (17.3)	122 (22.0)	84 (13.4)
101-200	868 (27.4)	119 (21.5)	182 (29.0)
201-300	454 (14.3)	33 (6.0)	115 (18.3)
>300	639 (20.2)	19 (3.4)	183 (29.1)
Teaching status			
Nonteaching hospital	2861 (90.3)	546 (98.4)	542 (86.3)
Teaching hospital	306 (9.7)	9 (1.6)	86 (13.7)
Profit status			
For-profit	671 (21.2)	126 (22.5)	123 (19.6)
Not for-profit	2502 (78.9)	433 (77.5)	505 (80.4)
Urban hospital			
Urban	2389 (75.3)	280 (50.1)	584 (93.0)
Excess readmission ratio, mean (SD)			
AMI	1.0010 (0.0543)	0.9946 (0.0474)	1.0053 (0.0521)
Heart failure	1.0015 (0.0728)	0.9953 (0.0624)	1.0068 (0.0711)
Pneumonia	1.0020 (0.0783)	0.9884 (0.0714)	1.0062 (0.0755)
COPD	1.0013 (0.0592)	0.9955 (0.0516)	1.0058 (0.0559)
THKA	1.0065 (0.1218)	0.9839 (0.1174)	1.0066 (0.1113)
CABG	1.0046 (0.1023)	0.9920 (0.0948)	1.0071 (0.0880)

Abbreviations: AMI, acute myocardial infarction; CABG, coronary artery bypass graft surgery; COPD, chronic obstructive pulmonary disease; MA, Medicare Advantage; THKA, total hip or knee arthroplasty.

^a^ Table provides descriptive statistics in 2019 for hospitals in first quintile of MA penetration, fifth quintile of MA penetration, and overall. Note that the number of hospitals in 2019 (n = 3165) is fewer than for the whole sample (n = 3203).

**Figure 2.**
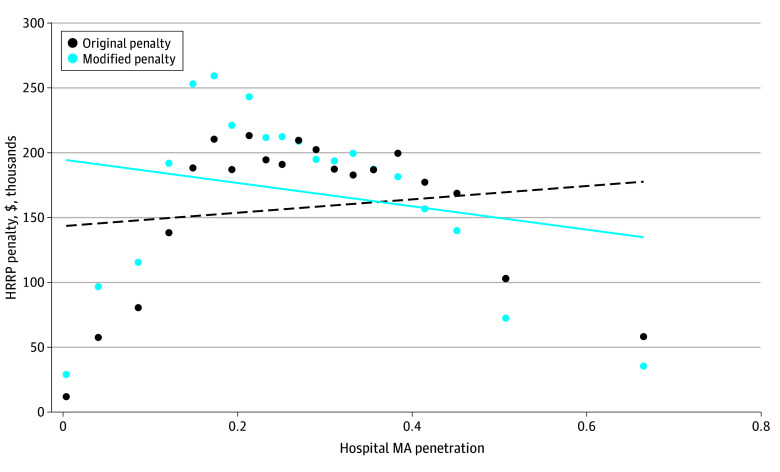
Original and Modified Penalties by Medicare Advantage Penetration, Under Nonpeer Grouping Methodology Figure illustrates differences in correlations between reported penalties (ie, original penalty) and penalties calculated from excess readmission ratios that account for Medicare Advantage (MA) penetration (ie, modified penalty). Each point corresponds to 1 of 20 equally sized groups of 598 hospitals binned according to level of MA penetration. The strength of association between MA penetration and penalty increases after accounting for MA penetration (ie, slope of line fitting points for modified penalty is steeper than slope of line fitting points for original penalty). This suggests that at lower levels of MA penetration (ie, with less unobserved selection), hospitals should have higher penalties, while at higher levels of MA penetration (ie, with more unobserved selection), hospitals should have lower penalties, relative to penalties calculated under the original penalty regime. The correlation coefficient for original penalty is 0.0288. The correlation coefficient for modified penalty is −0.0290.

Without peer grouping, higher MA penetration (ie, more unobserved selection) was associated with lower penalties, both in aggregate (Figure 2) and per discharge (eFigure 1 and eTable 3 in Supplement 1). While penalties were highest for hospitals in the second quintile of MA penetration under both original and rescaled measures of hospital performance (ERRs), higher MA penetration was associated with higher penalties using original ERRs but lower penalties using rescaled ERRs. Specifically, mean penalties using rescaled ERRs increased for hospitals in lower quintiles of MA penetration and decreased in higher quintiles. For example, mean penalties were $261 362.70 and $299 491.60 in quintile 2 and $201 394.00 and $188 200.80 in quintile 5 under original and rescaled measures, respectively.

After rescaling ERRs by MA penetration, we estimated that mean (SD) annual penalties would increase by $30 736 ($24 819.75) for hospitals in Q1 of MA penetration, $48 439 ($42 668.52) in Q2, $13 258 ($33 537.23) in Q3, and $2061 ($39 180.56) in Q4, and decrease by $26 915 ($42 017.23) in Q5, although we observed significant within-quintile variation in changes to penalties (eTable 4 in Supplement 1). Across hospitals, correcting for unobserved selection would result in annual penalty redistributions amounting to approximately $284 million.

**Figure 3.**
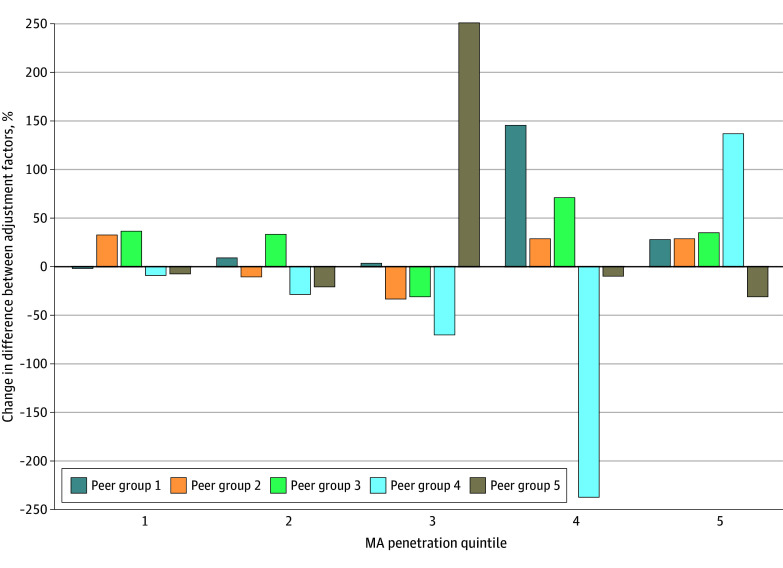
Association of Peer Grouping With Adjustment Factors After Adjusting for Medicare Advantage Penetration Graph shows the percentage change in the difference between adjustment factors that account for Medicare Advantage (MA) penetration and original adjustment factors under peer grouping relative to nonpeer grouping within quintiles of MA penetration. Lower peer groups have smaller shares of dually eligible beneficiaries, while higher peer groups have larger shares of dually eligible beneficiaries. Tabular results may be found in eTable 5 in Supplement 1.

Peer grouping did not attenuate changes in penalties driven by MA penetration. Rescaling ERRs by MA penetration resulted in similar changes in adjustment factors and penalties across MA penetration quintiles, with differences in magnitude of 2% to 11% under non–peer grouping and peer grouping paradigms (eTables 3 and 4 in Supplement 1). Across hospitals, correcting for unobserved selection under peer grouping would yield annual penalty redistributions amounting to approximately $297 million, 4.6% more than under non–peer grouping. When we investigated peer group-specific differences, no consistent patterns emerged (Figure 3 and eTable 5 in Supplement 1), although corrections tended to be smaller in lower quintiles (ie, less MA penetration) and larger in higher quintiles (ie, more MA penetration). To understand why peer grouping may have limited moderation over estimated adjustment factors and penalties, we examined the association between MA penetration and DE beneficiary share, which defines peer groups, and found only modest correlation (eFigure 2 in Supplement 1).

HRRP-targeted conditions were also studied separately. Significant increases in ERRs were observed among all HRRP conditions, except CABG and pneumonia. The percentage change in ERR after accounting for MA penetration was 0.59% for AMI, 1.3% for THKA, 0.32% for heart failure, and 0.13% for COPD (eTable 6 in Supplement 1).

## Discussion

We studied how accounting for unobservable patient severity in ERR estimation affects HRRP adjustment factors and penalties under both original non–peer grouping and current peer grouping methodologies. We found that explicit adjustment for MA penetration, a proxy for unobservable patient severity, would yield significant penalty redistributions totaling $284 million to $297 million annually, or $1.1 billion to 1.2 billion across our 4-year study period. These redistributions in aggregate would exceed 50% of total HRRP penalties and are 4 times larger than those from the 2019 shift to peer grouping.^23^

We observed that ERRs risk-adjusted according to current HRRP methods, which rely upon TM-only data, do not adequately account for favorable selection into MA. After rescaling observed ERRs for MA penetration and reestimating penalties (and adjustment factors), the association between MA penetration and penalties strengthens (ie, the magnitude of the slope increases). We interpreted this to mean that relatively conservative penalties were levied on hospitals with lower MA penetration and unobservably lower-risk TM beneficiaries, while excessive penalties were incurred by hospitals with higher MA penetration and unobservably higher-risk TM beneficiaries. These distortions likely came from unobservable differences in patient risk between MA and TM populations that vary by hospital (given greater unmeasured disease severity for TM patients in areas with higher MA penetration)^9,10,11,12^ that were not captured in HRRP’s risk adjustment methodology. Given the strong economic case for investments in readmission reductions, these distortions may skew hospital incentives for addressing performance issues.^24,25^

Although an area of debate,^8^ favorable selection into MA has been found to occur within hierarchical condition categories (HCCs),^26^ which claims-based classification CMS uses to risk-adjust MA reimbursements. HCCs include AMI and COPD, 2 HRRP conditions for which costs were overestimated, and thus, potentially favorable to MA.^27^ If HRRP risk adjustment captured additional information beyond HCCs, it is plausible that unobserved severity missed by HCCs could be captured observably by HRRP, in which case, our findings could reflect differences in readmissions from hospital quality rather than unobserved severity. However, HRRP risk adjustment relied upon condition categories, which were the same underlying classifications of diagnosis groups used in MA risk adjustment. Since risk adjustment methods were comparable between MA and HRRP, and because we controlled for quality measures, unobserved severity from MA likely persists in HRRP. Given care management differences across programs,^28^ it was also possible that favorable selection among MA vs TM enrollees may not apply after conditioning on inpatient hospitalizations. However, HCCs reflected inpatient and outpatient encounters, mitigating this concern.

We found evidence for these distortions under both non–peer grouping and peer grouping paradigms. After adjusting for MA penetration under non–peer grouping, penalties increased for hospitals in the first 4 quintiles of MA penetration and decreased for hospitals in the fifth quintile. Relative to non–peer grouping, accounting for MA penetration under peer grouping yielded marginally larger penalty corrections in the first 4 quintiles of MA penetration of 3% to 11% and smaller penalty corrections in the fifth quintile of 2%. Although we observed considerable within-quintile hospital-level heterogeneity that may reflect attenuated distortions for some hospitals, peer grouping did not mitigate distortions in aggregate.

Peer grouping is intended to address unmeasured social risk differences across hospitals^29^ and has been associated with large changes in hospital-level penalties.^15^ Peer group definitions are derived from the distribution of DE beneficiaries, with higher peer groups having higher DE share. If unmeasured social risk (proxied by DE share) were highly associated with unmeasured clinical risk (proxied by MA penetration), we should expect peer grouping to attenuate distortions estimated after accounting for MA penetration. However, because DE share is only modestly associated with MA penetration, peer grouping is insufficient for addressing unobserved selection.

We also considered whether peer grouping differentially addressed distortions for certain peer groups. We may expect such a pattern if correlations between DE share and unobserved severity differ in the patient populations served within different peer groups. However, no peer group had smaller corrections under peer grouping relative to non–peer grouping across quintiles of MA penetration.

Finally, we investigated heterogeneity by HRRP-targeted condition. Corrections were driven most by AMI and THKA and least by CABG and pneumonia. These results suggest that either unobserved severity differs among condition-specific patient populations, or condition-specific readmissions are differentially sensitive to unobserved severity.

### Policy Implications

CMS risk adjustment models, which exclusively rely upon TM data, cannot account for unobservable selection into MA. Resulting omitted variable bias creates distortions for incentive programs like HRRP that are based upon hospital-level risk adjustment. We propose 2 feasible solutions.

First, hospital-level MA penetration could be incorporated directly into current risk adjustment models. To the extent that MA penetration is associated with unobserved severity, that degree of selection would be accounted for explicitly. Such an approach may be feasible to implement in subsequent CMS risk adjustment model revisions. However, incorporating MA penetration directly into such a complicated model may prevent transparency about the role that MA penetration plays in risk adjustment, relative to other factors.

Second, hospital-level MA penetration could be incorporated into peer group definitions. Currently, peer group thresholds are determined by discretizing hospital DE beneficiary share into quintiles. While DE share is associated with socioeconomic status and associated health risks,^30^ peer grouping based only on DE share misses important measures of patient risk relevant to readmission performance.^16^ Furthermore, DE share is only modestly correlated with MA penetration. Taken together, our findings suggest that current peer grouping methods were insufficient to account for favorable selection into MA. Instead, peer groups could be defined based on the interaction of DE and MA penetration to account for socioeconomic and selection concerns, simultaneously. Peer grouping methods were generally favored in policy circles for transparency, although determining exact thresholds for peer groups is rather arbitrary, with potentially large implications for hospitals just on either side of these thresholds.

CMS has also issued a proposed rule for FY 2026 that would incorporate MA beneficiary data explicitly into calculations for risk-adjusted hospital readmission performance and decrease the HRRP lookback period from 3 to 2 years.^31^ If this policy were adopted, we may expect distortionary concerns from unobservable selection into MA to be reduced because performance measures should account for health distributions of both MA and TM populations. However, there were challenges to incorporating MA encounter data, given its relative incompleteness compared with TM claims and differences in coding practices that ultimately influence risk adjustment.^32^ Narrower lookback periods may also increase statistical noise in model estimates. Further work is necessary to understand reactions to, and spillovers from, such policies.

### Limitations

This study has limitations. First, rather than reconstruct ERRs with additional controls for MA penetration in HRRP’s hierarchical risk adjustment model, we instead rescaled observed ERRs using linear regression estimations. We assume that both approaches would provide similar estimates.^15,24,25^ Our method is also adaptable to the policy context. In HRRP’s current implementation, ERRs are estimated before peer group-specific performance standards are imposed to determine adjustment factors and penalties.^33,34^ Similarly, our strategy first leverages ERRs generated from HRRP’s hierarchical model, subsequently adjusts ERRs for MA penetration, and finally computes adjustment factors and penalties using these adjusted ERRs. As in HRRP, statistical significance does not bear any role in the calculation of these penalties.

Second, because we rescaled ERRs, we may have overadjusted for variables that are collinear with those already in the original ERR-generating process within the hierarchical risk adjustment model. Including collinear controls would yield overly conservative estimates, which suggests that our estimates of MA penetration on adjustment factors and penalties should be interpreted as lower bounds.

Third, because we only had MedPAR data through 2020, we assumed constant growth in hospital-level spending from 2019 to 2020 to extrapolate spending for 2021 and 2022. This may have underestimated penalties for some hospitals and overestimated them for others. We assumed that this linear extrapolation would not produce systematic bias on average.

Fourth, the use of MA penetration and hospital and county controls may not wholly capture the extent of unobserved selection in any given hospital. This measurement error should attenuate observed associations and again suggests that our estimates be interpreted as lower bounds.

Our analysis should not be interpreted causally. In case MA penetration were associated with quality (eg, if high-penetration hospitals were systematically lower quality with more excess readmissions), we control for known measures of hospital quality and only interpret MA penetration as a proxy for unobserved risk. Any other unobserved factors associated with quality and admissions could result in omitted variable bias, however, because these factors are likely associated with our controls, this bias would be mitigated. We do not suggest that differences in MA penetration are associated with adjustment factors or penalties.

## Conclusions

In this cohort study of hospitals exposed to HRRP from 2019 to 2022, we found that unobserved selection among TM beneficiaries may distort HRRP penalty calculations under both nonpeer grouping and peer grouping methods. Holding other factors constant, hospitals with smaller MA beneficiary shares receive more conservative penalties, while hospitals with greater shares incur excessive penalties. After accounting for MA penetration, we estimated penalty redistributions nearing $300 million annually and exceeding 50% of total collected HRRP penalties. Including MA penetration explicitly in risk adjustment or in peer group definitions may dampen distortions from unobservable patient severity in HRRP penalty calculations.

## Notes

Supplement 1. **eTable 1.** Fiscal Year to Calendar Year Crosswalk Across Datasets **eMethods 1. **Data Structure Alignment **eMethods 2.** Equations for Rescaling ERRs and Calculating Associated Adjustment Factors and Penalties **eTable 2.** Distribution of Change in Average Hospital-Level Medicare Advantage Penetration, 2019-2022 **eFigure 1.** Original and Modified Adjustment Factors by MA Penetration Under Non–Peer Grouping Methodology **eTable 3. **Change in Adjustment Factors after Rescaling ERRs With and Without Peer Grouping **eTable 4. **Change in Penalties After Rescaling ERRs With and Without Peer Grouping **eTable 5. **Change in Adjustment Factors by Peer Group After Rescaling ERRs With and Without Peer Grouping **eFigure 2. **Hospital-Year Association Between Dually Eligible and Medicare Advantage Beneficiary Shares **eTable 6.** Condition-Specific Change in Excess Readmission Ratio

Supplement 2.
Data Sharing Statement
