# Supplementary material for: Hospital Readmission Reduction Program Penalties for Hospitals With High Medicare Advantage Penetration
Source: JAMA Netw Open. 2026 Jan 22;9(1):e2554972. doi: 10.1001/jamanetworkopen.2025.54972 (PMC12828625; doi:10.1001/jamanetworkopen.2025.54972)
Supplement: Supplement 1. — eTable 1. Fiscal Year to Calendar Year Crosswalk Across Datasets eMethods 1. Data Structure Alignment eMethods 2. Equations for Rescaling ERRs and Calculating Associated Adjustment Factors and Penalties eTable 2. Distribution of Change in Average Hospital-Level Medicare Advantage Penetration, 2019-2022 eFigure 1. Original and Modified Adjustment Factors by MA Penetration Under Non–Peer Grouping Methodology eTable 3. Change in Adjustment Factors after Rescaling ERRs With and Without Peer Grouping eTable 4. Change in Penalties After Rescaling ERRs With and Without Peer Grouping eTable 5. Change in Adjustment Factors by Peer Group After Rescaling ERRs With and Without Peer Grouping eFigure 2. Hospital-Year Association Between Dually Eligible and Medicare Advantage Beneficiary Shares eTable 6. Condition-Specific Change in Excess Readmission Ratio [file jamanetwopen-e2554972-s001.pdf]

Supplemental Online Content

Chopra Z, Ryan AM, Hoffman GJ. Effects of Medicare Advantage on the hospital readmissions reduction program. *JAMA Netw Open*. 2026;9(1):e2554972. doi:10.1001/jamanetworkopen.2025.54972

eTable 1. Fiscal Year to Calendar Year Crosswalk Across Datasets
eMethods 1. Data Structure Alignment
eMethods 2. Equations for Rescaling ERRs and Calculating Associated Adjustment Factors and Penalties
eTable 2. Distribution of Change in Average Hospital-Level Medicare Advantage Penetration, 2019-2022
eFigure 1. Original and Modified Adjustment Factors by MA Penetration Under Nonpeer Grouping Methodology
eTable 3. Change in Adjustment Factors After Rescaling ERRs With and Without Peer Grouping
eTable 4. Change in Penalties After Rescaling ERRs With and Without Peer Grouping
eTable 5. Change in Adjustment Factors by Peer Group After Rescaling ERRs With and Without Peer Grouping
eFigure 2. Hospital-year Association Between Dually Eligible and Medicare Advantage Beneficiary Shares
eTable 6. Condition-Specific Change in Excess Readmission Ratio

This supplemental material has been provided by the authors to give readers additional information about their work.

© 2026 Chopra Z et al. *JAMA Network Open*.

eTable 1. Fiscal Year to Calendar Year Crosswalk across Datasets

Dataset	HRRP Supplemental Readmissions Files		SDoH	MedPAR		Impact File
Purpose	*Computing Excess Readmission Ratios*		*Contextual Factors*	*Hospital MA penetration*	*Penalty estimation*	*Hospital characteristics*
**FY / CY**	**Penalty Periods (FY)**[a]	**Performance Periods (FY)**[b]	**(CY)**[c]	**(CY)**[d]	**(CY)**	**(FY)**
	2019	FY 2015-2017	2015	2014-2017	2019	2019
	2020	FY 2016-2018	2016	2015-2018	2020	2020
	2021	FY 2017-2019	2017	2016-2019	2019-2020	2021
	2022	FY 2018-2020	2018	2017-2020	2019-2020	2022

[a] Year in which a penalty is imposed. Fiscal year (FY) corresponds to July 1st through June 30th, e.g., FY2019 is 7/1/ 2018 - 6/30/2019.
[b] Lookback years for which performance data is collected, e.g., the FY 2015-2017 period corresponds to 7/1/2014-6/30/2017.
[c] SDoH = Social Determinants of Health (from the Agency of Healthcare Research and Quality); CY data for SDoH were chosen to correspond to the approximate midpoint of the 3 fiscal-year period for each HRRP performance period.
[d] The calendar year (CY) definition of FY 2022 for MA and MedPAR files stops in December 2019 rather than June 2020 due to the COVID-19 pandemic, per CMS protocol.

© 2026 Chopra Z et al. *JAMA Network Open.*

eMethods 1. Data Structure Alignment

1. Fiscal Year (FY) 2019-2022 HRRP Supplemental Readmissions files used to determine:

- Condition-specific (e.g., AMI, heart failure) excess readmissions ratios (ERRs)
- Hospital-level admission volumes for each HRRP-targeted condition
- Peer group assignment (based on hospitals' proportions of dual eligibles)
- Peer group median ERR (to create rescaled ERRs using peer grouping methodology)

2. CY 2015-2018 AHRQ Social Determinants of Health (SDoH) files used to determine:

- County-year rate of home health agencies per 1,000 residents
- County-year percentage of residents ages 65 and older
- County-year percentage of residents never married
- County-year percentage of residents with less than high school education
- County-year percentage of residents unemployed
- County-year percentage of residents in poverty

3. Calendar Year (CY) 2014 - 2020 100% MedPAR claims used to determine:

- Hospital-level MA penetration using weighted volumes of MA and overall (MA + FFS) patient admissions during FY 2019-2022
- Hospital-level spending for CY 2019-2020 (to estimate original and modified penalties, using original and rescaled ERRs)
- Hospital-level spending for CY 2021-2022 (linearly extrapolated by applying CY 2019-2020 growth rate to future CYs)

4. FY 2019-2022 CMS Impact Files used to determine:

- Teaching hospital status (major teaching status is defined as a resident-to-bed ratio >= 0.25)
- Safety-net hospital status (safety-net status is defined as being in the top quartile of disproportionate share hospitals)
- Hospital bed size (categories defined as 1-50, 51-100, 101-200, 201-300, and 301+)

© 2026 Chopra Z et al. *JAMA Network Open.*

eMethods 2. Equations for Rescaling ERRs and Calculating Associated Adjustment Factors and Penalties

Estimating Risk-Adjusted ERRs:
We estimated models for each targeted condition (e.g., acute myocardial infarction), regressing the excess readmissions ratio (ERR) on hospital-year-level MA penetration ($MA\_Penetration_{it}$), time-varying hospital characteristics (X_{it}), time-varying county characteristics (X_{ct}), and county fixed effects (μ_c), for condition *c* in hospital *i* in county *j* in year *t*.

$$ERR_{cijt} = \beta_0 + \beta_1(MA\_Penetration_{cit}) + \beta_2(X_{cit}) + \beta_3(X_{cjt}) + \mu_{cj} + \varepsilon_{cijt}$$

Rescaling ERRs:
After estimating each model, we obtained predicted and expected ERRs for each condition *c* in hospital *i* in year *t*. Predicted ERRs were risk-adjusted ERRs determined for each condition-hospital-year. Expected ERRs were risk-adjusted ERRs determined for each condition-year across all hospitals. To obtain condition-hospital-year-specific ERRs that account for MA penetration, for each condition-hospital-year, we rescaled ERRs by subtracting predicted ERR from expected ERR and then adding this sum to the actual ERR:

$$Rescaled\ ERR_{cit} = ERR_{cit} + (Expected\ ERR_{ct} - Predicted\ ERR_{cit})$$

The rescaled, or corrected, ERR represents condition-specific performance (i.e., excess readmissions) when accounting for unobserved differences in the risk of hospital patient populations as proxied by hospital-level MA penetration.

Calculating Associated Adjustment Factors and Penalties:
To translate rescaled ERRs (that account for MA penetration) into adjustment factors and penalties, we followed CMS' HRRP calculations (see Tilson and Hoffman, 2012, p. 52). Specifically, we obtained the aggregate payments for excess readmissions, which equal the condition-specific payments for excess readmissions (i.e., for each condition for a given hospital, the product of the base operating payments, the number of admissions, and the ERR), summed across all conditions. Addressing Medicare Hospital Readmissions

We obtained the aggregate payments for excess readmissions (APER) under the original and modified (peer grouping) HRRP methodologies.

Original HRRP: $_{cit} = \sum_{c=1,\ldots,6}(Base\ payment_{cit} * Admission\ count_{cit} * max\{(ERR_{cit} - 1.0), 0)$

Modified HRRP: $APER\ _{cit} = \sum_{c=1,\ldots,6}(Base\ payment_{cit} * Admission\ count_{cit} * max\{(ERR_{cit} - Medican\ peer\ group\ ERR_{cit}), 0)$

We obtained the excess readmissions payment ratio, ERPR, by calculating 1 minus the ratio of aggregate payments for excess readmissions to the aggregate payments for all discharges.

© 2026 Chopra Z et al. *JAMA Network Open*.

$$ERPR_{cit} = 1 - \left(\frac{APER_{cit}}{Aggregate\ payments\ for\ all\ discharges_{cit}}\right)$$

Methods for determining adjustment factors and penalties proceed similarly under peer grouping. However, when determining the aggregate payments for excess readmissions, ERRs are adjusted by subtracting the ERR of the median peer group from the ERR of any given hospital (as opposed to comparing the hospital-specific ERR to the national average ERR, which, by definition, is equal to 1).

$$\text{Peer grouping } ERR_{cit} = ERR_{cit} - Median\ peer\ group\ ERR_{ct}$$

We obtained the adjustment factor (AF), which is the scalar used for adjusting hospital payments, by calculating 1 minus ERPR and imposing a 3% penalty cap.

$$AF_{cit} = 1 - max\{0.03, ERPR_{cit}\}$$

© 2026 Chopra Z et al. *JAMA Network Open.*

eTable 2. Distribution of Change in Average Hospital-level Medicare Advantage Penetration, 2019-2022

Penalty Year (FY)	Mean	Min.	Max	25th percentile	75th percentile	Interquartile Range (IQR)
2019	0.25	0.0	0.95	0.14	0.35	0.21
2020	0.26	0.0	0.95	0.25	0.36	0.21
2021	0.27	0.0	0.95	0.15	0.36	0.21
2022	0.30	0.0	0.95	0.19	0.40	0.20

Penalty years correspond to earlier performance periods, e.g., penalty year 2019 corresponds to a performance period of FY 2015-2017. Hospital-level Medicare Advantage (MA) penetration is assessed during the respective performance periods.

© 2026 Chopra Z et al. *JAMA Network Open.*

eFigure 1. Original and Modified Adjustment Factors by MA Penetration under Non-peer Grouping Methodology

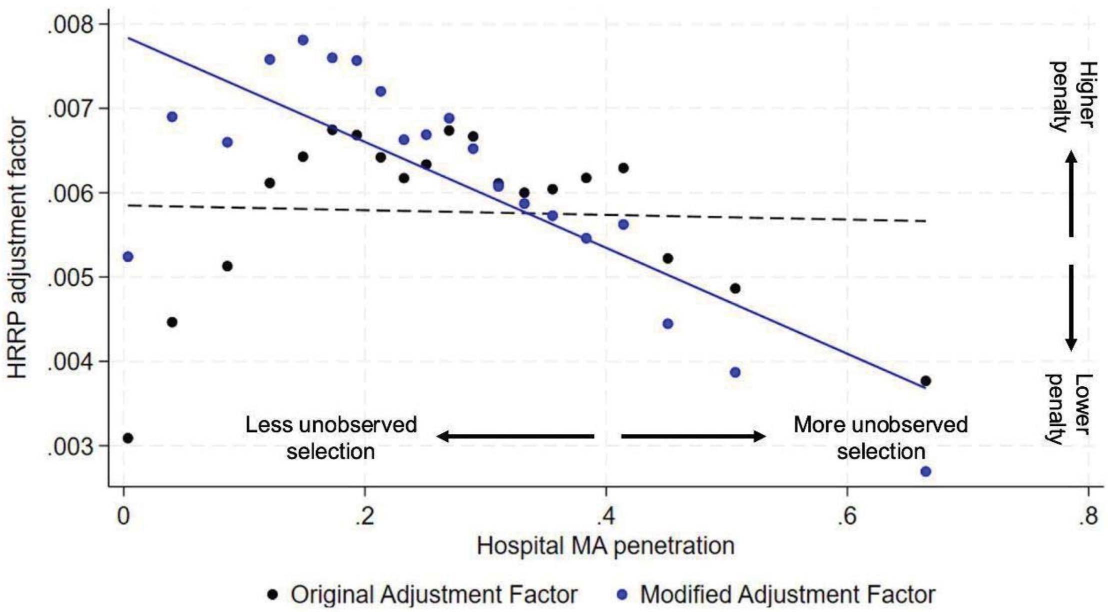

Figure shows differences in correlations between reported adjustment factors ("original adjustment factor") and adjustment factors calculated from excess readmission ratios that account for MA penetration ("modified adjustment factor"). Adjustment factors may be interpreted as changes in per-discharge penalties. Each point corresponds to 1 of 20 equally sized groups of 598 hospitals binned according to level of MA penetration. The strength of association between MA penetration and adjustment factor (where a higher adjustment factor indicates a larger HRRP penalty) increases after accounting for MA penetration (i.e., slope of line fitting points for "modified adjustment factor" is steeper than slope of line fitting points for "original adjustment factor"). This suggests that at lower levels of MA penetration (i.e., with less unobserved selection), hospitals should have higher penalties, while at higher levels of MA penetration (i.e., with more unobserved selection), hospitals should have lower penalties, relative to penalties calculated under the "original adjustment factor" regime. The correlation coefficient for "original adjustment factor" is -0.0166. The correlation coefficient for "modified adjustment factor" is -0.1460.

© 2026 Chopra Z et al. *JAMA Network Open.*

eTable 3. Change in Adjustment Factors after Rescaling ERRs with and without Peer Grouping

Non-Peer Grouping	Hospital Count	Mean Δ(AF)	SD Δ(AF)	25th % Δ(AF)	50th % Δ(AF)	75th % Δ(AF)
MA Penetration Q1	1,929	0.0020	0.0047	0	0.0004	0.0027
MA Penetration Q2	2,476	0.0011	0.0030	-0.0002	0.0004	0.0020
MA Penetration Q3	2,543	0.0003	0.0024	-0.0006	0	0.0012
MA Penetration Q4	2,588	-0.0002	0.0025	-0.0011	-0.0000	0.0005
MA Penetration Q5	2,428	-0.0009	0.0024	-0.0016	-0.0004	0

Peer Grouping	Hospital Count	Mean Δ(AF)	SD Δ(AF)	25th % Δ(AF)	50th % Δ(AF)	75th % Δ(AF)
MA Penetration Q1	1,929	0.0021	0.0049	0	0.0005	0.0027
MA Penetration Q2	2,476	0.0011	0.0030	-0.0002	0.0005	0.0020
MA Penetration Q3	2,543	0.0004	0.0024	-0.0006	0.0000	0.0012
MA Penetration Q4	2,588	-0.0002	0.0025	-0.0011	0	0.0006
MA Penetration Q5	2,428	-0.0009	0.0024	-0.0016	-0.0003	0.0000

% Change of Peer Grouping relative to Non-Peer Grouping	Mean Δ(AF)	Median Δ(AF)
MA Penetration Q1	5.8%	21.3%
MA Penetration Q2	2.4%	12.4%
MA Penetration Q3	11.2%	NA
MA Penetration Q4	-8.7%	100.0%
MA Penetration Q5	-2.0%	-4.9%

Table shows changes in adjustment factors (AF) at the mean, median, 1st quartile, and 3rd quartile under non-peer grouping and peer grouping after accounting for MA penetration as well as percent differences in mean and median AF changes under peer grouping relative to non-peer grouping.

© 2026 Chopra Z et al. *JAMA Network Open.*

eTable 4. Change in Penalties after Rescaling ERRs with and without Peer Grouping

Non-Peer Grouping	Hospital Count	Mean Δ(Penalties)	SD Δ(Penalties)	Total Δ(Penalties)	25th % Δ(Penalties)	50th % Δ(Penalties)	75th % Δ(Penalties)
MA Penetration Q1	1,929	30736.39	104117.3	59290496.31	0	2870.72	24819.75
MA Penetration Q2	2,476	48439.36	201523.3	119935855.36	-3202.01	5490.21	39466.51
MA Penetration Q3	2,543	13257.7	101451.6	33714331.1	-12663.84	0	20873.39
MA Penetration Q4	2,588	2060.91	163069	5333635.08	-29247.15	-48.93	9933.41
MA Penetration Q5	2,428	-26914.52	74344.2	-65348454.56	-42017.23	-5694.33	0

Peer Grouping	Hospital Count	Mean Δ(Penalties)	SD Δ(Penalties)	Total Δ(Penalties)	25th % Δ(Penalties)	50th % Δ(Penalties)	75th % Δ(Penalties)
MA Penetration Q1	1,929	32872.61	106906.7	63411264.69	0	3457.03	26422.56
MA Penetration Q2	2,476	50090.52	201266.1	124024127.52	-2776.57	6043.11	41084.19
MA Penetration Q3	2,543	14684.26	103638.1	37342073.18	-12131.56	401.91	22811.45
MA Penetration Q4	2,588	3128.63	166423.9	8096894.44	-28932.67	0	10684.42
MA Penetration Q5	2,428	-26373.9	75780.84	-64035829.2	-41665.09	-5361.87	522.10

% Change of Peer Grouping relative to Non-Peer Grouping	Mean Δ(Penalties)	Median Δ(Penalties)
MA Penetration Q1	7.0%	20.4%
MA Penetration Q2	3.4%	10.1%
MA Penetration Q3	10.8%	NA
MA Penetration Q4	5.2%	100.0%
MA Penetration Q5	-2.0%	-5.8%

Table shows changes in penalties at the mean, median, 1st quartile, and 3rd quartile under non-peer grouping and peer grouping after accounting for MA penetration as well as percent differences in mean and median penalty changes under peer grouping relative to non-peer grouping.

© 2026 Chopra Z et al. *JAMA Network Open.*

eTable 5. Change in Adjustment Factors by Peer Group after Rescaling ERRs with and without Peer Grouping

Non-Peer Grouping	Peer Group 1	Peer Group 2	Peer Group 3	Peer Group 4	Peer Group 5
MA Penetration Q1	0.0037	0.0019	0.0017	0.0019	0.0012
MA Penetration Q2	0.0014	0.0012	0.0007	0.0012	0.0009
MA Penetration Q3	0.0003	0.0003	0.0003	0.0006	0.0000
MA Penetration Q4	-0.0003	-0.0005	-0.0003	0.0004	-0.0004
MA Penetration Q5	-0.0012	-0.0010	-0.0009	-0.0005	-0.0010

Peer Grouping	Peer Group 1	Peer Group 2	Peer Group 3	Peer Group 4	Peer Group 5
MA Penetration Q1	0.0036	0.0026	0.0023	0.0017	0.0011
MA Penetration Q2	0.0016	0.0011	0.0010	0.0009	0.0007
MA Penetration Q3	0.0004	0.0002	0.0002	0.0002	0.0001
MA Penetration Q4	-0.0008	-0.0006	-0.0005	-0.0005	-0.0003
MA Penetration Q5	-0.0015	-0.0013	-0.0012	-0.0011	-0.0007

% Change	Peer Group 1	Peer Group 2	Peer Group 3	Peer Group 4	Peer Group 5
MA Penetration Q1	-1.7%	32.4%	36.1%	-8.7%	-7.3%
MA Penetration Q2	8.9%	-10.3%	33.0%	-28.7%	-20.9%
MA Penetration Q3	3.6%	-33.2%	-30.8%	-70.2%	1939.3%
MA Penetration Q4	144.9%	28.3%	70.9%	-236.9%	-9.5%
MA Penetration Q5	27.7%	28.2%	34.6%	137.0%	-31.1%

Table shows mean changes in adjustment factors (AF) by peer group under non-peer grouping and peer grouping after accounting for MA penetration as well as percent differences in mean AF changes by peer group under peer grouping relative to non-peer grouping.

© 2026 Chopra Z et al. *JAMA Network Open.*

eFigure 2. Hospital-year Association between Dually Eligible and Medicare Advantage Beneficiary Shares

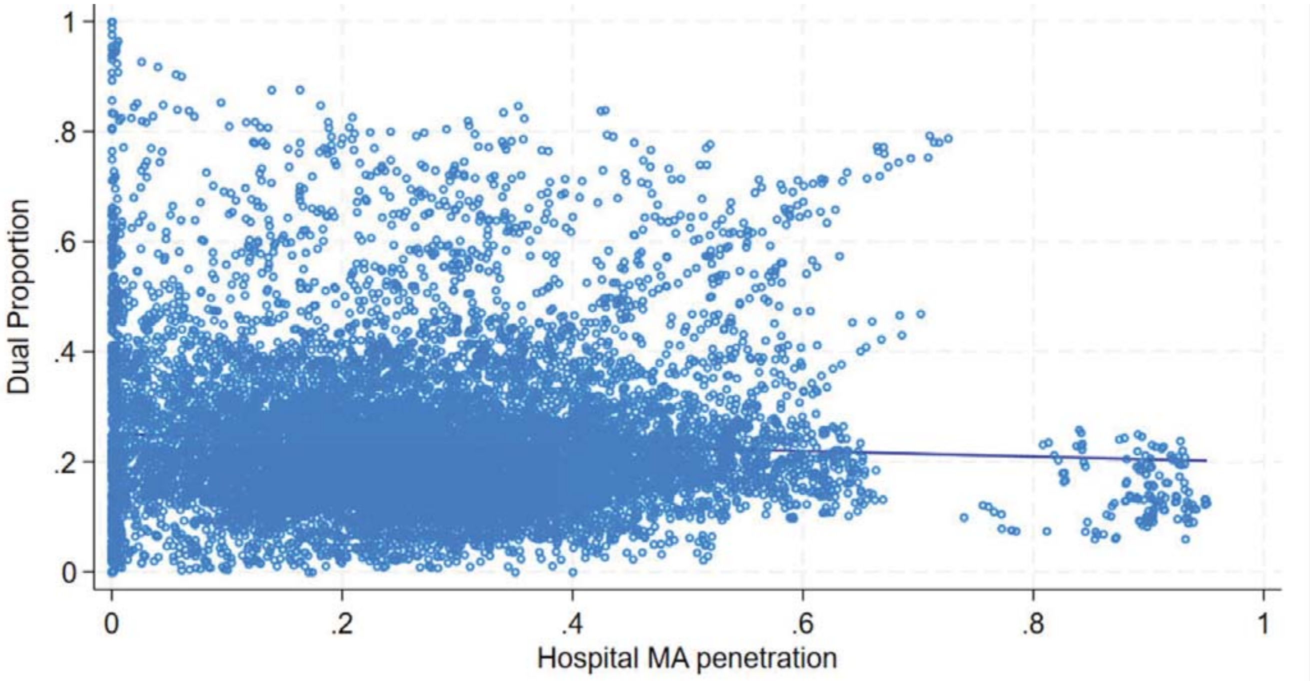

Figure shows correlation between share of dually eligible (dua; DEI) beneficiaries and Medicare Advantage (MA) beneficiaries at the hospital-year level. The correlation coefficient is -0.0598.

© 2026 Chopra Z et al. *JAMA Network Open.*

eTable 6. Condition-specific Change in Excess Readmission Ratio

HRRP-targeted Condition	Baseline Mean ERR	Change in ERR	Percent Change in ERR	p-value
Acute Myocardial Infarction	1.0017	−0.0059	0.59%	<0.001
Heart Failure	1.0020	−.0032	0.32%	<0.001
COPD	1.0014	0.0013	0.13%	0.015
Total Hip/Knee Arthroplasty	1.0075	−0.0134	1.33%	<0.001
Pneumonia	1.0019	0.0014	0.14%	1
CABG	1.0022	−0.0025	0.25%	0.18

Table shows condition-specific excess readmission ratios (ERRs) prior to accounting for MA penetration ("baseline mean ERR"), the level change in ERRs after accounting for MA penetration ('change in ERR"), the percent change in ERRs, defined as the ratio of "change in ERR" to "baseline mean ERR," and p-values from tests of significance for the level change in ERRs.

© 2026 Chopra Z et al. *JAMA Network Open.*
