# Supplementary material for: Hospital Readmission Reduction Program Penalties for Hospitals With High Medicare Advantage Penetration
Source: JAMA Netw Open. 2026 Jan 22;9(1):e2554972. doi: 10.1001/jamanetworkopen.2025.54972 (PMC12828625; doi:10.1001/jamanetworkopen.2025.54972)
Supplement: Supplement 2. — Data Sharing Statement [file jamanetwopen-e2554972-s002.pdf]

Data Sharing Statement

Chopra. Medicare Advantage and the Hospital Readmissions Reduction Program. *JAMA Netw Open*. Published January 22, 2026. doi:10.1001/jamanetworkopen.2025.54972

Data
Data available: No
